# The enhanced anticoagulation for graphene induced by COOH^+^ ion implantation

**DOI:** 10.1186/s11671-014-0705-2

**Published:** 2015-01-27

**Authors:** Xiaoqi Liu, Ye Cao, Mengli Zhao, Jianhua Deng, Xifei Li, Dejun Li

**Affiliations:** Energy & Materials Engineering Centre, College of Physics and Materials Science, Tianjin Normal University, Tianjin, 300387 China

**Keywords:** Ion implantation, Graphene, Carboxyl, Anticoagulation

## Abstract

Graphene may have attractive properties for some biomedical applications, but its potential adverse biological effects, in particular, possible modulation when it comes in contact with blood, require further investigation. Little is known about the influence of exposure to COOH^+^-implanted graphene (COOH^+^/graphene) interacting with red blood cells and platelets. In this paper, COOH^+^/graphene was prepared by modified Hummers' method and implanted by COOH^+^ ions. The structure and surface chemical and physical properties of COOH^+^/graphene were characterized by scanning electron microscopy (SEM), X-ray photoelectron spectroscopy (XPS), and contact angle measurement. Systematic evaluation of anticoagulation, including *in vitro* platelet adhesion assays and hemolytic assays, proved that COOH^+^/graphene has significant anticoagulation. In addition, at the dose of 5 × 10^17^ ions/cm^2^, COOH^+^/graphene responded best on platelet adhesion, aggregation, and platelet activation.

## Background

Graphene, the thinnest two-dimensional (2D) allotrope of carbon, is a novel nanomaterial with a single sheet of carbon atoms packed in a perfect honeycomb structure [1,2]. It is attracting a lot of attention due to its high surface area, high thermal conductivity, fast charged carrier mobility, and strong Young's modulus [2,3]. In the last years, there has been a surge in research work on graphene and graphene-based materials (GBMs), with applications in fields as diverse as nanoelectronics, energy technology, sensors, composite materials, energy conversion and storage, electrocatalysis, and electronics [2,4]. Recently, graphene has been proposed for biomedical applications such as bioassays, biosensing/bioimaging, drug delivery, anticancer therapy, electrical stimulation of cells, antibacterial materials, catalysis, adsorption of enzyme, and cell imaging [4-7]. Furthermore, approaches for applications in biomedical engineering, regenerative medicine, and biotechnology are under study [4].

Enhancement of the surface properties of medical implants for improved integration into their biological environment is a major focus of current biomaterial research [8]. Ion implantation, which enables one to inject any element or group into the near-surface region of any solid [9], provides a practical way to get functional surface with stability and reproducibility [10-12]. The method modifies the structure of a target-near-surface by bombardment by ions [11]. Because of these advantages, ion implantation for surface modification of multiwalled carbon nanotubes (MWCNTs) has been used in our previous works [13,14]. However, in comparison with MWCNTs, graphene as a single-layer two-dimensional material composed of layers of carbon atoms forming six-membered rings presents long and reactive edges which make it more accessible to doping and hold more excellent properties from surface area to electronic [15]. So in this paper, COOH^+^ ions were implanted into graphene, instead of MWCNTs, to achieve a better anticoagulant material. Except toxicological and ecological risks, anticoagulation is a prerequisite for the use of graphene in biological or medical applications. It is necessary to evaluate the anticoagulant risks of graphene [4]. Rabbit and rat blood was selected for probing the above questions in this study.

Some previous investigation demonstrated that the anticoagulation of MWCNTs was greatly influenced by surface functional groups. In our previous work, we focused on cell and blood compatibility of MWCNTs by different functional groups [16-20]. The improved anticoagulation was observed in COOH^+^ implantation-MWCNTs when compared with pristine MWCNTs [13,16]. The method of COOH^+^ implantation introduced functional carboxyl groups into the surfaces of materials. The existence of hydroxy and other oxygen-containing functions was thought to be able to induce the hydrophilicity of MWCNTs [13,21]. To graphene, thus, appropriate COOH^+^ implantation could also enable it to exhibit attractive anticoagulation [22]. In the present investigation, firstly, the COOH^+^/graphene shows better anticoagulation than pristine graphene for platelets and red blood cells. In addition, the content of carboxyl group in COOH^+^/graphene affected its anticoagulation. Compared with our earlier works, the ion implantation in this study is in great dose and high purity. The findings presented in this study also throw light into that COOH^+^/graphene may serve as an excellent candidate material for potential biomedical applications in various areas.

## Methods

### Preparation of samples and characteristic analysis

The graphene powders were produced according to modified Hummers' method [23-26]. Details for preparing the COOH^+^/graphene were as follows: in the first step, graphene was ultrasonically dispersed in an organic solvent called 1-methyl-2-pyrrolidinone (NMP) for several hours to make it dissolved evenly. The concentration of graphene was 0.5 mg/cm^2^. Then, the graphene solution was sprayed by airbrush with Ar flow on the heated (100°C) SiO_2_ wafers which were used as support materials for graphene. Next, baking was done for 3 h (250°C) under argon protection to remove the organic solvent completely [23,27]. Finally, COOH^+^ ions were implanted on graphene using a Kaufman-100 implanter (Tongchuang Applied Plasma Technology Center, Chengdu, China). The COOH^+^ was generated from methanoic acid, which would be ionized by heating in water bath and bombardment of accelerated electrons in ion source [9,11,13]. These ions were accelerated by a high tension onto the graphene samples. During implantation, the ion energy was 20 keV [28]. The beam current density was controlled under 119 μA/cm^2^ [12,29], and the work pressure in the target chamber was kept at 1.5 × 10^−2^ Pa. The different doses of COOH^+^/graphene (1 × 10^17^, 5 × 10^17^, and 1 × 10^18^ ions/cm^2^) were obtained by adjusting the irradiation time.

The surface hydrophilicity of graphene and COOH^+^/graphene was analyzed with sessile drop method. Deionized water was dropped onto the sample surface, and images were recorded using the CAM KSV021733 optical contact-angle inclinometer (Nunc, Finland). The process lasted for ten times, and the results came from the average of these data. Scanning electron microscope (SEM) imaging was performed using a field-emission scanning electron microscope (FESEM; Hitachi S-3000N, Hitachi, Ltd., Chiyoda, Tokyo, Japan). X-ray photoelectron spectroscopic (XPS) measurements were performed on a Kratos Axis Ultra Al (alpha) X-ray photoelectron spectrometer (Kratos Analytical, Manchester, UK).

### Platelet adhesion assays

Platelet-rich plasma (PRP) was prepared by centrifuging the fresh blood with anticoagulant at 1,200 rpm for about 15 min. The fresh blood was withdrawn from a healthy rat. Additionally, PRP was put immediately into 24-well culture plate which all the samples were placed in them. The 24-well culture plates were placed immediately in the incubator at 37°C for 30 min. The samples were then fixed in glutaraldehyde, dried with critical point drying, and plated for examination of SEM.

### Hemolysis assays

**Figure 1:**
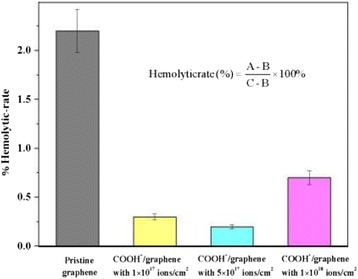
**Hemolytic rate results.** Hemolytic rates of pristine graphene, COOH^+^/graphene with 1 × 10^17^ ions/cm^2^, COOH^+^/graphene with 5 × 10^17^ ions/cm^2^, and COOH^+^/graphene with 1 × 10^18^ ions/cm^2^.

In order to investigate the hemolysis of pristine graphene and COOH^+^/graphene *in vitro*, we measured the absorption of free hemoglobin (Hb) at 545 nm in the plasma. The samples were immersed in the physiological salt water and placed in the water bath at 37°C for 30 min. The diluted blood (anticoagulant blood with 2% potassium oxalate 4:5 normal saline) was successively added into the solution of all groups. After incubation at 37°C for 60 min, the blended liquid was centrifuged at 1,000 rpm for 5 min. The supernatants were transferred into the cuvettes, and the optical density (OD) values were measured at 545 nm with a spectrophotometer. Hemolysis ratio was calculated by the formula in Figure 1, where *A*, *B*, and *C* are the average absorbance of samples, negative controls, and positive controls, respectively [30]. All data were expressed as means ± SD for five measurements. The error bars on graphs reflecting ± SD.

## Results and discussion

**Figure 2:**
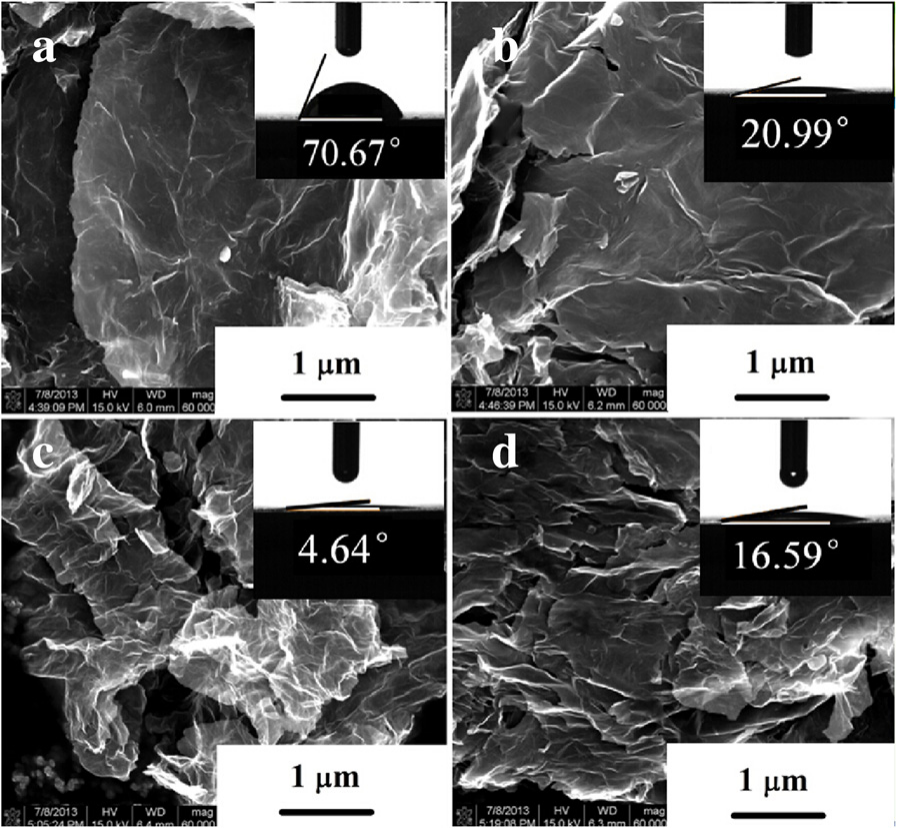
**SEM and CA images of pristine graphene and COOH** ^**+**^ **/graphene. (a)** Pristine graphene, **(b)** COOH^+^/graphene with 1 × 10^17^ ions/cm^2^, **(c)** COOH^+^/graphene with 5 × 10^17^ ions/cm^2^, and **(d)** COOH^+^/graphene with 1 × 10^18^ ions/cm^2^.

Figure 2 depicts a high-resolution SEM image indicating the surface morphology (wavy structure) of graphene and COOH^+^/graphene. The transparent nature of the folded crystalline layers is clearly shown in this figure. Graphene and COOH^+^/graphene exhibit typical wrinkled structure, indicating the high surface/volume ratio and two-dimensional structure of graphene. And COOH^+^/graphene show more wrinkles with the COOH^+^ dose increasing. The most remarkable effect of carboxyl ion implantation also can be seen in the contact angle measurements as shown in Figure 2. The droplet on the graphene irradiated with doses at 1 × 10^17^ ions/cm^2^ leaves a contact angle of 20° ± 2°. COOH^+^/graphene with 5 × 10^17^ ions/cm^2^ leaves a contact angle of 4° ± 2°. COOH^+^/graphene with 1 × 10^18^ ions/cm^2^ leaves a contact angle of 16° ± 2°. But the droplet on pristine graphene's surface remains highly spherical with the angle of 70° ± 2°, which implies that the pristine graphene is more hydrophobic than COOH^+^/graphene. As we know, graphene as well as other carbon nanostructures are naturally hydrophobic, so surface wettability is highly improved by COOH^+^ ion implantation. And the materials' surfaces are rich in oxygen after carboxyl ion implantation. Surface hydrophilia may have an effect on anticoagulation property because the initial phase of connection affects the contacts between blood and materials through various forces and adsorbing sundry proteins [3]. COOH^+^/graphene is an oxygen-containing graphene derivative with partial breakage of sp^2^-sp^2^ bonds into sp^3^-sp^3^ bonds for inserting some pendent groups (hydroxy, epoxy, and carboxylic). These functional groups facilitate the interaction between the host solution and implanted samples. It gives us much inspiration to realize that COOH^+^ ion implantation may bring great advantages for biomaterials to improve their anticoagulation properties [31].

**Figure 3:**
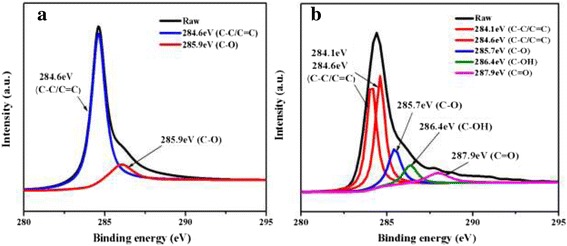
**C1s XPS spectra of (a) pristine graphene and (b) COOH** ^**+**^ **/graphene.**

XPS is a powerful tool to study the presence and the content of surface functional groups on the samples [32-34]. The C1s XPS spectra of pristine graphene and COOH^+^/graphene are presented in Figure 3. By analysis of binding energy (BE) values, the feature of binding between carbon and oxygen is confirmed. The C1s band can be fitted to several components, corresponding to carbon atoms in different positions: C-C/C = C, C-O, C = O, and C-OH. The XPS C1s spectra for graphene and COOH^+^/graphene are different as displayed in the figure. COOH^+^/graphene shows distinguished COOH^+^ signal. The oxygen-related functional groups such as hydroxyl (C-O), carbonyl (C = O), and hydroxy (C-OH) were present in COOH^+^/graphene, as O content increases from 6.97 atm.% for pristine graphene (Graphene of 0 atm.% oxygen content could not be produced by modified Hummers' method. The graphene used in this study had very low oxygen content, which could be called pristine graphene [35].) to 10.73 atm.% for COOH^+^/graphene with 1 × 10^17^ ions/cm^2^, 10.77 atm.% for COOH^+^/graphene with 5 × 10^17^ ions/cm^2^, and 11 atm.% for COOH^+^/graphene with 1 × 10^18^ ions/cm^2^ after COOH^+^ ion implantation. These results indicate the presence of oxygen element in functionalized graphene. In contrast, the COOH^+^ signal for the pristine graphene is not observable. Whereas the appearance of C = O and C-OH peaks after COOH^+^ ion implantation reveals the cleavage of some pendant such as C-C and C = C after COOH^+^ ion implantation. The above result verifies the COOH^+^ ion implantation effect in graphene, which may assist their dispersion in aqueous solution.

**Figure 4:**
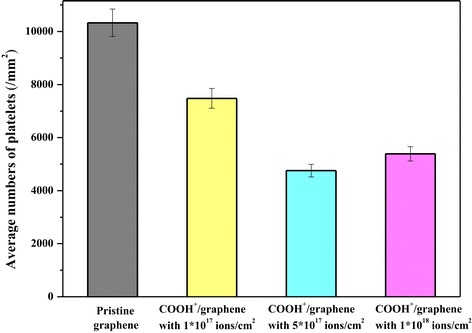
**Statistical results of the platelets adhered to pristine graphene and COOH** ^**+**^ **/graphene.** The number of platelets on pristine graphene, COOH^+^/graphene with 1 × 10^17^ ions/cm^2^, COOH^+^/graphene with 5 × 10^17^ ions/cm^2^, and COOH^+^/graphene with 1 × 10^18^ ions/cm^2^.

**Figure 5:**
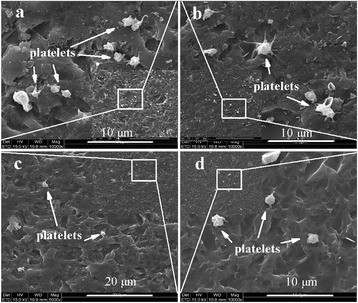
**SEM images of the platelet adhesion testing. (a)** Pristine graphene, **(b)** COOH^+^/graphene with 1 × 10^17^ ions/cm^2^, **(c)** COOH^+^/graphene with 5 × 10^17^ ions/cm^2^, and **(d)** COOH^+^/graphene with 1 × 10^18^ ions/cm^2^.

*In vitro* platelet adhesion testing is carried out to study the quantity, morphology, aggregation, and pseudopodium of the adherent platelets [36]. The statistical results of the platelets adhered to pristine graphene and COOH^+^/graphene are shown in Figure 4, representing the average of ten measurements, and the data are translated into platelet numbers in ‘per unit area’. Platelet numbers adhering on graphene decrease as the following order: pristine graphene > COOH^+^/graphene with 1 × 10^17^ ions/cm^2^ > COOH^+^/graphene with 1 × 10^18^ ions/cm^2^ > COOH^+^/graphene with 5 × 10^17^ ions/cm^2^. It is clear that the number of platelets on the COOH^+^/graphene is lower than that on the pristine graphene, and the number of adhered platelets decreases by COOH^+^ ion implantation in graphene, which performs preferable anti-adhesive capacity for COOH^+^/graphene. The value of COOH^+^/graphene with 5 × 10^17^ ions/cm^2^ is even lower than that of COOH^+^/graphene with 1 × 10^18^ ions/cm^2^. Comprehensively considering the results of both platelet adhesion and water contact angle assays, the platelet numbers adhering to the surface reduce obviously as surface wettability increases. The enhanced wettability of both functionalized graphene results in the reduction of platelet adhesion, which may lead to the improvement in anticoagulation. Figure 5 shows the morphology of blood platelets exposed to different materials. Platelets adhered on all graphenes remain discrete and discoidal shape. However, it is obvious through the SEM image that more deformed platelets with pseudopodia are present on pristine graphene surface than COOH^+^/graphene, as shown in Figure 5a, which implies that COOH^+^/graphene has much better anticoagulation than pristine graphene. Nevertheless, COOH^+^/graphene show slightly thrombogenicity at a fluence of 1 × 10^18^ ions/cm^2^. Thrombogenicity occurring on the material surface in contact with blood is a complicated process. The surface free energy has an important effect on the process, particularly for some hydrophilic polymers that show less platelet activation. Compared with pristine graphene, COOH^+^/graphene with 1 × 10^17^ ions/cm^2^, 1 × 10^18^ ions/cm^2^, and 5 × 10^17^ ions/cm^2^ shows the lowest platelet aggregation and activation, although some platelets exhibit the slight activation (Figure 5c). Given the above, it is implied that the COOH^+^/graphene with 5 × 10^17^ ions/cm^2^ provides further evidence for its anticoagulation.

The *in vitro* hemolysis test, by detecting the concentration of free hemoglobin in the serum after interaction with pristine graphene and COOH^+^/graphene, indicated that significant hemolysis (more than 5% [37,38]) cannot be detected in all the samples [39]. The descriptive statistics of hemolytic rate results are indicated in Figure 1. The hemolysis ratio is 2% to 5% and 0% to 2%. It can be seen that the hemolysis grade is slightly hemolytic and non-hemolytic [30]. Slight hemolysis can only be detected in pristine graphene. Other samples present almost undetectable hemoglobin concentration [39]. And after the interaction with the anticoagulated blood and following centrifugation, the negative control solution in the centrifuge tube forms two layers, the upper layer of clearly colorless supernatant and the under layer of whole erythrocytes; the positive control solution performs a red supernatant on account of the broken red blood cells (RBCs); both pristine graphene solution and COOH^+^/graphene solution forms colorless supernatant same as the negative control group. However, pristine graphene and COOH^+^/graphene show transparent supernatants like the negative control group due to the undamaged RBC. These results indicate that pristine graphene and COOH^+^/graphene with three ion doses are all nonhemolytic materials.

## Conclusions

We have shown here that functionalized graphene was successfully acquired by COOH^+^ ion implantation while microstructural aspects, microstructure, and valence bond were systematically investigated. The interaction of red blood cells and platelets with pristine graphene and COOH^+^/graphene were investigated and compared detailedly. Three kinds of COOH^+^/graphene were shown to exhibit lower platelet adhesion, aggregation, and platelet activation than pristine graphene under the same conditions, especially for the COOH^+^/graphene with 5 × 10^17^ ions/cm^2^. No significant toxicity effects could be found on COOH^+^/graphene. The COOH^+^/graphene showed an observable improvement in anticoagulation as the COOH^+^ ion was implanted. This study demonstrated superior anticoagulation of COOH^+^/graphene with 5 × 10^17^ ions/cm^2^ as promising and effective biomedical material for biomaterial industry.

## Abbreviations

COOH^+^/graphene: COOH^+^-implanted graphene
MWCNTs: multiwalled carbon nanotubes
PRP: platelet-rich plasma
OD: optical density
BE: binding energy
RBCs: red blood cells
