# Erratum: The Enhanced Anticoagulation for Graphene Induced by COOH+ ion Implantation

**DOI:** 10.1186/s11671-015-1014-0

**Published:** 2015-09-04

**Authors:** Xiaoqi Liu, Ye Cao, Mengli Zhao, Jianhua Deng, Xifei Li, Dejun Li

**Affiliations:** Energy & Materials Engineering Centre, College of Physics and Materials Science, Tianjin Normal University, Tianjin, 300387 China

After the publication of this work [1], we noticed an error whereby the images of Figs. 1, 2, 3 and 5 were interchanged, and therefore do not correspond to their legends. They are interchanged and therefore do not correspond to their legends. The original version of this article was corrected. The corrected figures are given below:

**Fig. 1:**
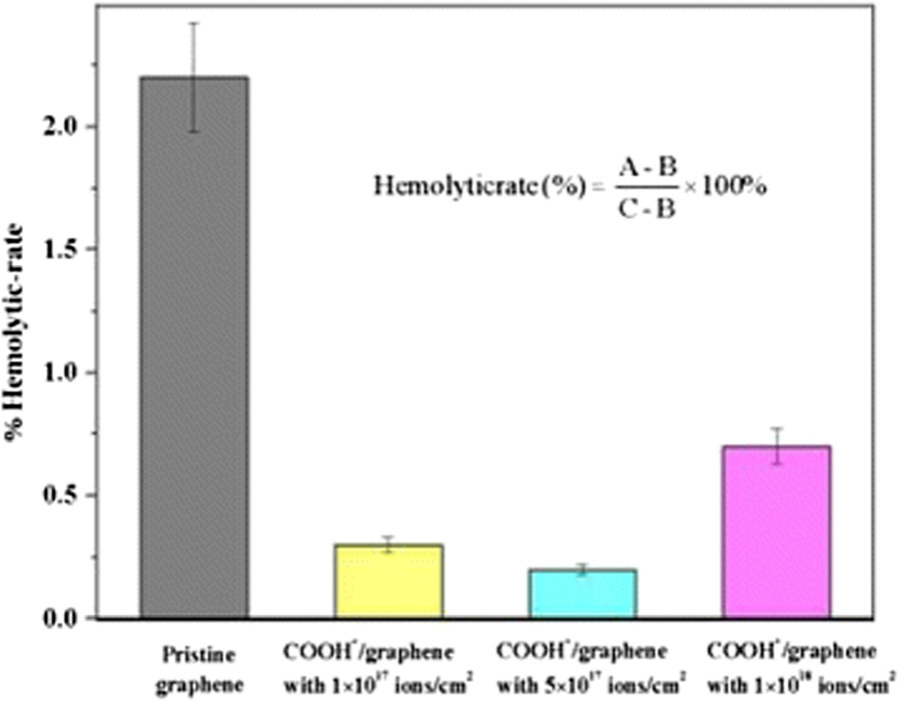
Hemolytic rate results. Hemolytic rates of pristine graphene, COOH+/graphene with 1 × 1017 ions/cm2, COOH+/graphene with 5 × 1017 ions/cm2, and COOH+ /graphene with 1 × 1018 ions/cm2

**Fig. 2:**
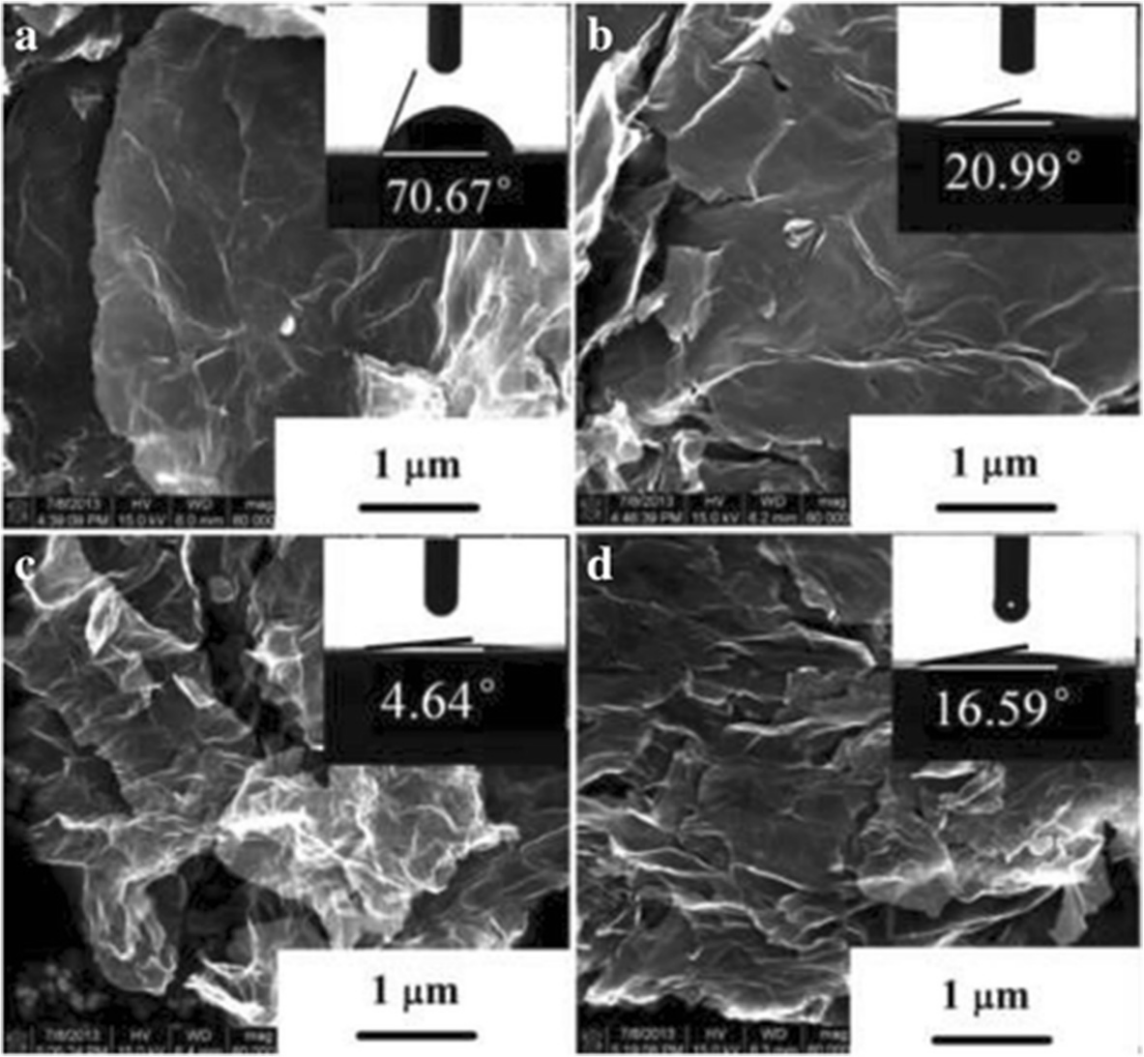
SEM and CA images of pristine graphene and COOH+/graphene. (**a**) Pristine graphene, (**b**) COOH+/ graphene with 1 × 1017 ions/cm2, (**c**) COOH+/graphene with 5 × 1017 ions/cm2, and (**d**) COOH+/graphene with 1 × 1018 ions/cm2

**Fig. 3:**
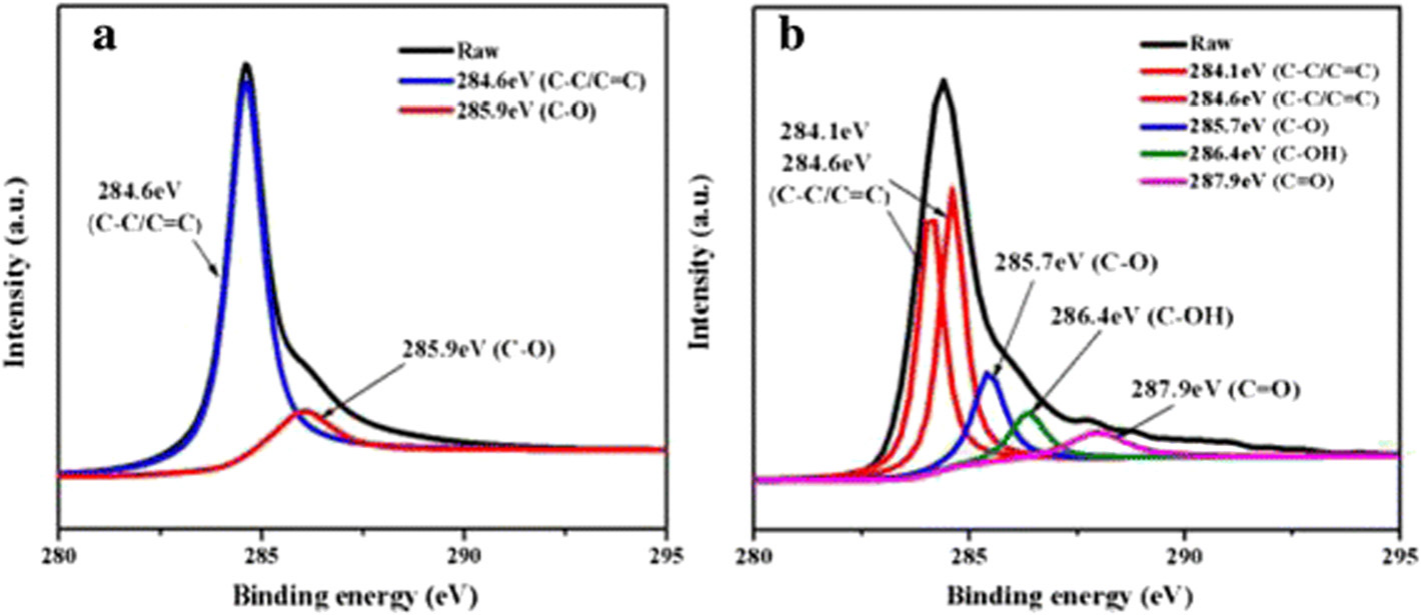
C1s XPS spectra of (**a**) pristine graphene and (**b**) COOH+/graphene

**Fig. 5:**
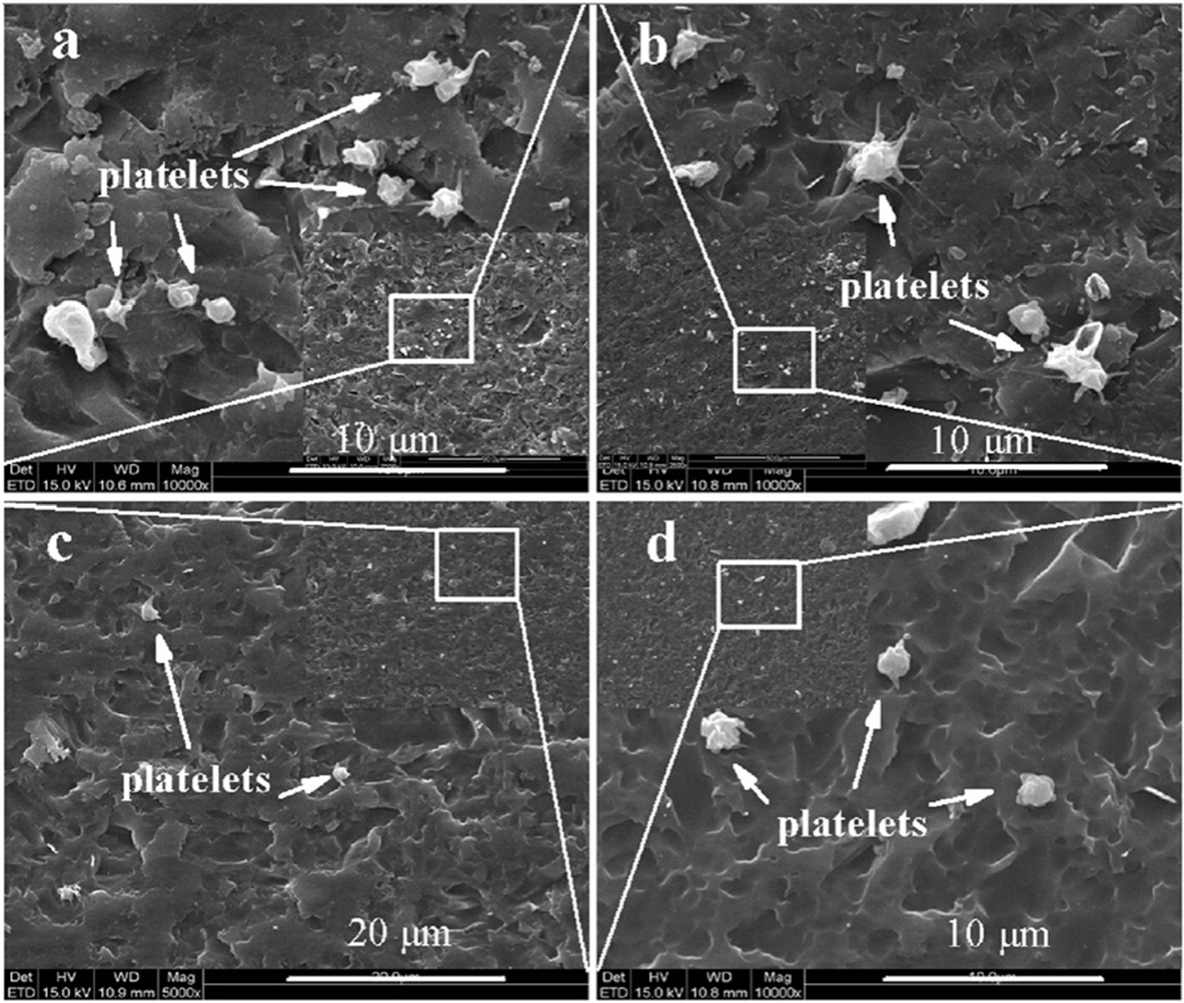
SEM images of the platelet adhesion testing. (**a**) Pristine graphene, (**b**) COOH+/graphene with 1 × 1017 ions/cm2, (**c**) COOH+/graphene with 5 × 1017 ions/cm2, and (**d**) COOH+/graphene with 1 × 1018 ions/cm2
